# Editors’ Note

**DOI:** 10.63144/ijt.2025.6728

**Published:** 2025-12-12

**Authors:** Ellen R. Cohn, Jana Cason

## Journal Overview

The International Journal of Telerehabilitation (IJT) is a biannual journal dedicated to advancing telerehabilitation by disseminating peer-reviewed information about current research and practices. IJT is indexed by PubMed and Scopus. IJT is published via the open journal system (OJS) and is now sponsored by the Hawaiʻi Pacific University, Graduate College of Health Sciences. This institutional support enables IJT to be subscription-free and require no author fees.

IJT enjoys a diverse international audience due to the expanding global relevance of telemedicine, telehealth, and telerehabilitation. In this issue, we are honored to present original work from: Australia, Brazil, Canada, India, New Zealand, the Netherlands, United States, and Ukraine. Articles from countries featured in past issues include Australia, Brazil, Canada, Colombia, Egypt, Germany, India, Indonesia, Italy, Malaysia, the Netherlands, Poland, Portugal, Saudi Arabia, Taiwan, Thailand, Ukraine, and the United States.

We are grateful to our expert peer reviewers who donate their time and hail from multiple disciplines and countries. Their suggestions invariably elevate the work of our most experienced authors. Our reviewers are not named herein, to preserve the anonymity of their reviews.

## New Beginnings

This is the second issue since IJT was “rehomed” to Hawaiʻi Pacific University, Graduate College of Health Sciences, where Dr. Jana Cason is now a full-time faculty member. We are grateful for the warm welcome from IJT’s new publisher, Sabrina Thomas, EdD, Director of Library and Learning Commons, Hawaiʻi Pacific University (HPU) and HPU’s capable team. Coincidentally, the IJT co-editors’ primary residences in the United States have recently been rehomed: Ellen Cohn to Florida, and Jana Cason to Alabama.

As well, we acknowledge Joseph K. Ruffing at the University of Pittsburgh who designed the first IJT journal website and article template in 2008 that continues to visually define the Journal, and colleagues at the Office of Scholarly Communication and Publishing at University of Pittsburgh Library System (ULS) whose work has been foundational to the journal. Their work is preserved in the University of Pittsburgh Library System (ULS) library system archive, as well as on the Hawaiʻi Pacific University journal archive.

## Next Issue: telerehab.hpu.edu

All former issues and the current issue are now located at: telerehab.hpu.edu. The next issue will be published in June 2026. IJT will open for new submissions on or about January 12, 2026, through March 1, 2026.

Me ka mahalo nui (With much gratitude),

Jana Cason, DHSc, OTR/L, FNAP, FAOTA

Ellen R. Cohn, PhD, CCC-SLP, ASHA-F, ATA F, NAP-F

IJT Co-Editors-in-Chief

